# Supplementary material for: The Impact of Malnutrition on Chronic Obstructive Pulmonary Disease (COPD) Outcomes: The Predictive Value of the Mini Nutritional Assessment (MNA) versus Acute Exacerbations in Patients with Highly Complex COPD and Its Clinical and Prognostic Implications
Source: Nutrients. 2024 Jul 17;16(14):2303. doi: 10.3390/nu16142303 (PMC11279935; doi:10.3390/nu16142303)
Supplement: Supplementary file 1 [file nutrients-16-02303-s001.zip › nutrients-3089696-supplementary.pdf]

nutrients

MDPI

**Supplementary Materials**

**Supplementary Table S1.** Multivariate regression results for mMRC (model 1), CAT (model 2), BI (model 3) and EQ-5D-3L (model 4), with counfonding variables.

| | *Model 1.1* | *Model 1.2* | *Model 2.1* | *Model 2.2* | *Model 3.1* | *Model 3.2* | *Model 4.1* | *Model 4.2* |
|---|---|---|---|---|---|---|---|---|
| $R^2$<br>$aR^2$ | 0.273<br>0.235 | 0.292<br>0.254 | 0.220<br>0.179 | 0.275<br>0.237 | 0.173<br>0.129 | 0.238<br>0.198 | 0.222<br>0.181 | 0.314<br>0.277 |
| | *β coefficient* | | | | | | | |
| Age | 0.023* | 0.022* | 0.100 | 0.095 | −0.279 | −0.271 | −0.013 | −0.011 |
| Gender (Male) | 0.181 | 0.213 | −0.515 | −0.214 | 2.063 | 1.429 | 0.387 | 0.304 |
| GOLD Class 2 | 0.647* | 0.675* | 3.879 | 4.158* | 0.489 | −0.183 | −0.324 | −0.405 |
| GOLD Class 3 | 1.02*** | 1.03*** | 6.096** | 6.134** | −12.05* | −11.48* | −1.060 | −1.048 |
| GOLD Class 4 | 1.27** | 1.197* | 6.079 | 5.310 | 3.329 | 5.309 | −0.019 | 0.209 |
| MNA-Short Form | −0.135*** | - | −1.075*** | - | 1.424* | - | 0.273*** | - |
| MNA-Total Score | - | −0.095*** | - | −0.844*** | - | 1.605*** | - | 0.231*** |

* $p<0.05$; ** $p<0.01$; *** $p<0.001$; An increase of 1 point is intended for continuous variables, except for model 4 (0.1-point increase); mMRC: modified Medical Research Council; CAT: COPD Assessment Test; $R^2$: coefficient of determination; $aR^2$: adjusted $R^2$; MNA: Mini Nutritional Assessment; EQ-5D-3L: EuroQol 5D 3 level.

**Supplementary Table S2A.** Competiting risks regression model for subjects "at risk of malnutrition" or "malnourished" according to the MNA-Short Form with confounding variables.

| | **SHR** | **Standard Error** | **z** | ***p*** | **95% Confidence Interval** |
|---|---|---|---|---|---|
| **Age** | 0.979 | 0.015 | −1.36 | 0.173 | 0.949-1.009 |
| **Gender (Male)** | 1.058 | 0.333 | 0.18 | 0.855 | 0.571-1.961 |
| **GOLD Class 2** | 1.084 | 0.657 | 1.10 | 0.894 | 0.330-3.556 |
| **GOLD Class 3** | 1.307 | 0.805 | 0.43 | 0.664 | 0.390-4.373 |
| **GOLD Class 4** | 1.812 | 1.394 | 0.77 | 0.440 | 0.401-8.186 |
| **CAT ≥ 10** | 1.566 | 0.641 | 1.10 | 0.273 | 0.702-3.494 |
| **At risk of malnutrition** | 3.768 | 2.060 | 2.43 | 0.015 | 1.290-11.004 |
| **Malnourished** | 6.120 | 3.518 | 3.15 | 0.002 | 1.984-18.880 |

SHR: sub-Hazard Ratio; MNA: Mini Nutritional Assessment; mMRC: modified Medical Research Council; CAT: COPD Assessment Test.

**Supplementary Table S2B.** Competiting risks regression model for subjects "at risk of malnutrition" or "malnourished" according to the MNA-Total Score with confounding variables.

| | SHR | Standard Error | z | *p* | 95% Confidence Interval |
|---|---|---|---|---|---|
| **Age** | 0.979 | 0.015 | −1.29 | 0.197 | 0.949-1.011 |
| **Gender (Male)** | 1.138 | 0.357 | 0.41 | 0.680 | 0.614-2.106 |
| **GOLD Class 2** | 1.261 | 0.737 | 0.40 | 0.691 | 0.401-3.963 |
| **GOLD Class 3** | 1.518 | 0.886 | 0.72 | 0.475 | 0.483-4.767 |
| **GOLD Class 4** | 2.623 | 1.919 | 1.32 | 0.188 | 0.624-11.012 |
| **CAT ≥ 10** | 1.729 | 0.715 | 1.32 | 0.186 | 0.768-3.893 |
| **At risk of malnutrition** | 3.084 | 1.244 | 2.79 | 0.005 | 1.399-6.801 |
| **Malnourished** | 4.643 | 2.356 | 3.03 | 0.002 | 1.717-12.552 |

SHR: sub-Hazard Ratio; MNA: Mini Nutritional Assessment; mMRC: modified Medical Research Council; CAT: COPD Assessment Test.
